# Vertical sleeve gastrectomy associates with airway hyperresponsiveness in a murine model of allergic airway disease and obesity

**DOI:** 10.3389/fendo.2023.1092277

**Published:** 2023-02-28

**Authors:** Jack T. Womble, Mark D. Ihrie, Victoria L. McQuade, Akhil Hegde, Matthew S. McCravy, Sanat Phatak, Robert M. Tighe, Loretta G. Que, David D’Alessio, Julia K. L. Walker, Jennifer L. Ingram

**Affiliations:** ^1^ Division of Pulmonary, Allergy and Critical Care Medicine, Department of Medicine, Duke University School of Medicine, Durham, NC, United States; ^2^ School of Nursing, Duke University, Durham, NC, United States; ^3^ Diabetes/Rheumatology Units, King Edward Memorial Hospital, Pune, India; ^4^ Division of Endocrinology, Metabolism and Nutrition, Department of Medicine, Duke University School of Medicine, Durham, NC, United States

**Keywords:** asthma, obesity, sleeve gastrectomy, bariatric surgery, airway hyperresponsiveness, airway inflammation, mouse model

## Abstract

**Introduction:**

Asthma is a chronic airway inflammatory disease marked by airway inflammation, remodeling and hyperresponsiveness to allergens. Allergic asthma is normally well controlled through the use of beta-2-adrenergic agonists and inhaled corticosteroids; however, a subset of patients with comorbid obesity experience resistance to currently available therapeutics. Patients with asthma and comorbid obesity are also at a greater risk for severe disease, contributing to increased risk of hospitalization. Bariatric surgery improves asthma control and airway hyperresponsiveness in patients with asthma and comorbid obesity, however, the underlying mechanisms for these improvements remain to be elucidated. We hypothesized that vertical sleeve gastrectomy (VSG), a model of metabolic surgery in mice, would improve glucose tolerance and airway inflammation, resistance, and fibrosis induced by chronic allergen challenge and obesity.

**Methods:**

Male C57BL/6J mice were fed a high fat diet (HFD) for 13 weeks with intermittent house dust mite (HDM) allergen administration to induce allergic asthma, or saline as control. At week 11, a subset of mice underwent VSG or Sham surgery with one week recovery. A separate group of mice did not undergo surgery. Mice were then challenged with HDM or saline along with concurrent HFD feeding for 1-1.5 weeks before measurement of lung mechanics and harvesting of tissues, both of which occurred 24 hours after the final HDM challenge. Systemic and pulmonary cytokine profiles, lung histology and gene expression were analyzed.

**Results:**

High fat diet contributed to increased body weight, serum leptin levels and development of glucose intolerance for both HDM and saline treatment groups. When compared to saline-treated mice, HDM-challenged mice exhibited greater weight gain. VSG improved glucose tolerance in both saline and HDM-challenged mice. HDM-challenged VSG mice exhibited an increase in airway hyperresponsiveness to methacholine when compared to the non-surgery group.

**Discussion:**

The data presented here indicate increased airway hyperresponsiveness in allergic mice undergoing bariatric surgery.

## Introduction

Asthma is a heterogenous chronic airway inflammatory disease impacting roughly 300 million people worldwide (1) and over 25 million people in the United States (2). Each year around 250,000 adults in the US are diagnosed with asthma and comorbid obesity (3, 4), a condition known to be poorly controlled by currently available pharmacologic therapies, contributing to increased hospitalization, poor asthma management, and decreased quality of life (5–9). Asthma in adults with comorbid obesity may be characterized by distinct endotypes involving different immune mechanisms (10). Generally, asthma which is diagnosed early in life is associated with high type 2 immune responses and an allergic clinical phenotype (5). Obesity contributes to the severity of allergic asthma (11) and corticosteroid insensitivity (12), complicating effective treatment of patients. Asthma onset following the development of obesity in adulthood is more prevalent in females and associated with a non-allergic phenotype and low type 2 immune responses (5, 13). Current projections estimate that 48.9% of the US population will experience obesity by 2030 (14). The rising prevalence of both asthma and obesity will necessitate a better understanding of disease development and care.

Allergic asthma is classically defined by allergen-induced airway inflammation and is represented by elevated serum immunoglobin E (IgE), type 2 cytokine (interleukins-4, -5, and -13 [IL-4, IL-5, IL-13]) production, and sputum eosinophilia (>1%-3%) (15, 16). The type 2 cytokine response drives airway inflammation, hyperresponsiveness (AHR), and remodeling which manifests as increased sub-epithelial fibrosis, mucus hypersecretion, and hyper-reactivity to environmental allergens (17). Although allergic asthma is commonly well controlled with beta-2-adrenergic agonists and inhaled corticosteroids (18), obesity significantly impairs therapeutic efficacy (7–9); however, the underlying mechanism(s) of this therapeutic impairment is poorly understood.

Despite the lack of effective standard and biologic therapies for asthma patients with comorbid obesity, weight loss through bariatric surgery is associated with improved asthma control and maintenance (19). A study conducted by Santos et al. found individuals who underwent bariatric surgery experience improved lung capacity, dynamic lung volumes and total respiratory resistance, with greater improvement occurring in patients with asthma and comorbid obesity (20). Additionally, a review of weight loss through surgical and non-surgical intervention identified bariatric surgery patients to have greater decreases in medication use, hospitalization, AHR, and improved lung function (21); benefits which occur as soon as 30 days post-operative and are sustained for at least 3 years (22).

Bariatric surgery may be modeled in obese rodents using vertical sleeve gastrectomy (VSG) procedures, as reviewed in (23, 24). These models have low mortality and may closely resemble the human surgical procedures in that post-operative outcomes may be observed that allow for evaluation of the physiological effects of bariatric surgery in obese mice and rats. Obesity in rodents may be induced using high fat, high carbohydrate or Western “cafeteria” diets (25), which stimulates significantly altered immune responses (26), gut microbiota changes (27) and inherent airway hyperresponsiveness (28). When combined with acute or chronic allergic challenge, models of obesity demonstrate that marked changes to pulmonary inflammation, remodeling and responsiveness occur that are distinct from changes induced by allergen exposures alone (26).

While studies have identified improvements in asthma exacerbation and control with bariatric surgery, the underlying physiological changes are poorly understood. This study reports the effects of VSG in a mouse model of obese allergic asthma. We hypothesized that VSG would improve glucose tolerance and airway inflammation, resistance, and fibrosis induced by chronic allergen challenge in obese mice. Surprisingly, and contrary to our hypothesis, our data show that in this model, VSG augmented allergen-induced airway resistance. A better understanding of how modeling of bariatric surgery in rodent modulates experimental asthma responses may provide insights regarding the impact of bariatric surgery in asthma and comorbid obesity.

## Methods

### Animals

Five-week-old male C57BL/6J mice were purchased from Jackson Laboratory. Animal care and experimental protocols were approved by the Duke University Institutional Animal Care and Use Committee and carried out in accordance with the American Association for the Accreditation of Laboratory Animal Care guidelines. Male C57BL/6J mice were used for experiments as they are more susceptible to diet-induced obesity than female C57BL/6J mice (29). All mice were housed in pathogen free facilities at Duke University. At week 0, 10, and 12 of the protocol, mice were tested for oral glucose tolerance and weighed.

### Oral glucose tolerance test

Mice were fasted for 5 hours prior to the glucose tolerance test. A bolus of 10% glucose solution (200 μL) was administered by oral gavage. The mice were restrained, and blood was collected from the tail vein. Blood was collected prior to gavage and at 10-, 30-, and 90-minutes post-gavage. Glucose levels were determined by an Accu-Chek Performa (Roche) blood glucose meter.

### Diet and treatments

**Figure 1:**
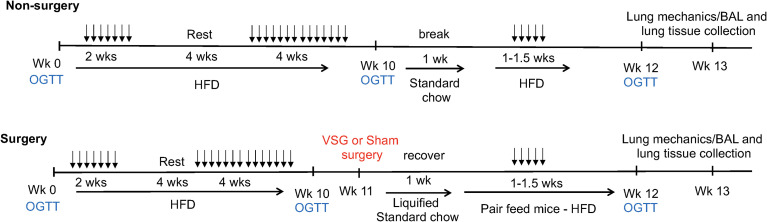
Schematic depiction of experimental time course. 5-week-old mice underwent oral glucose tolerance testing (OGTT) on weeks (Wk) 0, 10 and 12. 50 μg of house dust mite extract or saline (vehicle control) was delivered *via* intranasal administration in 40 μLs saline 3 times per week (doses shown as arrows) for the durations shown. High fat diet (HFD – 60% kcal fat) or standard chow diet (13% kcal fat) was given *ad libitum* for the durations shown. Vertical sleeve gastrectomy (VSG) or Sham surgeries were performed at week 11. Mice were pair fed after surgery. Lung mechanics measurements as well as bronchoalveolar lavage (BAL) and tissue collection in mice occurred at week 13.

Mice were fed a high-fat diet (HFD – 60% kcal fat, Research Diets #D12492i) for 10 weeks to induce obesity. A separate group of mice were fed a standard chow diet (normal chow – 13% kcal fat) for the same time frame. For the first 2 weeks, HFD-fed mice received intranasal phosphate-buffered saline (saline) or house dust mite (HDM) allergen (Greer Laboratories, XPB70D3A2.5, lots #360924 (Endotoxin 872.5 EU/vial) and #369446 (Endotoxin 1542.5 EU/vial), 50 µg of protein delivered in 40 µl PBS (30)) 3 days per week under light isoflurane anesthesia for allergen sensitization. After a 4-week break, in which mice continued to consume HFD, intranasal administration of PBS or HDM continued 3 days per week for 4 weeks. On week 11, mice underwent vertical sleeve gastrectomy (31) (VSG; n= 8 per group) or sham surgery (31) (n= 9 - 10 per group) followed by a 1-week recovery and were pair fed a liquified standard chow diet (powdered chow mixed with water). A third group of mice (n=11) did not undergo surgery (Non-surgery, NS) and were rested for 2 weeks and fed standard chow to match the surgery group. After recovery, all mice were then placed back on HFD and administered intranasal saline or HDM 3 days per week for 1-1.5 weeks (**Figure 1**).

### Surgery

Surgeries were performed as described in Douros et al. (32) Prior to VSG or Sham surgery, mice were fasted overnight. Mice were anesthetized under isoflourane and a roughly 1.5 cm midline incision was made below the xyphoid process. The suspensory ligament was incised, and the spleen was separated from the stomach. A Ligaclip (LS400, Ethicon) was placed on the stomach at the angle of His, forming a tube between the esophagus and pylorus, separating the majority of the stomach which was then excised. The Ligaclip was attached with three sutures through the stomach walls, and then the excision was closed with a continuous suture. Sham surgeries were identical to VSG except the stomach was not clipped and excised: a midline incision was made, the suspensory ligament was incised, and the stomach was separated from the spleen. The stomach was removed temporarily and then placed back in the abdomen and the incision was closed.

### Lung mechanics measurements

Airway responsiveness to intravenous methacholine was measured 24 hours after the final HDM exposure using a computer-controlled small animal ventilator (FlexiVent, Scireq) as previously described (33). The resulting impedance signal was used to calculate Newtonian resistance (R_n_), total respiratory system resistance (R_tot_), elastance (E), tissue damping (G), and tissue elastance (H). Central airway and total respiratory sensitivity (provocative concentration of methacholine resulting in a doubling of baseline airway resistance [PC_100_] and reactivity [K]) were calculated using non-linear regression analysis with exponential growth curve fit of the methacholine dose-response curve for each animal (34). Briefly, the data points in the dose response to methacholine underwent non-linear regression analysis with exponential growth curve fit. In doing so, dose response data were linearized. Thus, the linear curve fits the equation Y = mX + b. The slope (m) of the linear curve represents “K” (reactivity). PC100 is the bronchoconstrictor provocative concentration that causes a 100% increase (doubling) in baseline resistance.

### Bronchoalveolar lavage (BAL)

Immediately following lung mechanics measurements, lungs were lavaged with 1 mL saline 3 times. BAL fluid cells were separated by centrifugation, and cells were attached to slides using a Cytospin 3 Cytocentrifuge (ThermoFisher), fixed and stained with Easy III (Azer Scientific). Differential cell counts were obtained by counting 200 total cells under 200x magnification.

### Lung histology

Tissues were harvested immediately after BAL. Lungs were inflated to 25 cmH_2_O, fixed in 4% paraformaldehyde, and embedded in paraffin. Sections were stained with hematoxylin and eosin (H&E), Masson’s trichrome, and periodic acid-Schiff (PAS). Histological scoring was performed as described in Ihrie et al. (35) H&E sections were scored 0-4 for peribronchial inflammation, including depth and circumference of inflammatory cells in 10 circular airways per mouse lung section. PAS-stained sections were scored 0-4 for positive staining of airway epithelial layer mucus in 10 airways per mouse lung section (35). For both H&E and PAS, a score of 0 indicates little-to-no inflammation or mucus, respectively, and a score of 4 indicates severe inflammation or mucus, respectively. Scores for H&E staining were recorded across the 10 airways and the circumference and depth parameters were averaged together to determine the mean H&E score per mouse. For PAS staining, scores were recorded across the 10 airways to determine the mean PAS score per mouse. Peribronchial Masson’s trichrome staining was quantified by color thresholding in ImageJ in 9-10 airways per mouse lung section (35).

### mRNA quantification in mouse lung tissue

During harvest, the right middle lung lobe was immediately placed into TRI Reagent (MilliporeSigma). Lungs were then homogenized, and total RNA was isolated *via* standard procedure according to manufacturer’s instructions. RNA concentration was measured on a NanoDrop ND-1000 (ThermoFisher) and cDNA was prepared using the Applied Biosystems High-Capacity Reverse Transcription Kit. Quantitative real-time polymerase chain reaction (qRT-PCR) was then performed using Applied Biosystems TaqMan Gene Expression Master Mix and TaqMan primers (glyceraldehyde-3 phosphate dehydrogenase [*Gapdh*] Mm99999915_g1, elastin [*Eln*] Mm00514670_m1, collagen type 1 alpha 1 chain [*Col1a1*] Mm00801666_g1, collagen type 1 alpha 2 chain [*Col1a2*] Mm00483888_m1, glucagon-like peptide-1 receptor [*Glp1r*] Mm00445292_m1, interleukin-13 receptor alpha 1 [*Il13ra1*] Mm01302068_m1, interleukin-13 receptor alpha 2 [*Il13ra2*] Mm00515166_m1, interleukin-4 receptor alpha [*Il4ra*] Mm01275139_m1, transforming growth factor beta 1 [*Tgfb1*] Mm01178820_m1, mucin 5AC [*Muc5ac*] Mm01276718_m1, and mucin 5B [*Muc5b*] Mm00466391_m1). Fold change was calculated with the delta Ct method using the saline/NS as the control treatment, and *Gapdh* as the endogenous control.

### Enzyme-linked immunosorbent assay (ELISA)

Leptin (DY498), IL-13Rα2 (DY539), total (EMIGHE) and HDM-specific (3037) IgE, IL-13 (DY413), IL-5 (DY405), and total and active TGF-β1 (DY1679), were measured in serum, BAL fluid or homogenized lung tissue by ELISA using kits purchased from R&D systems or ThermoFisher Scientific. For lung tissue homogenates, total protein was determined using bicinchoninic acid (BCA) assay (Pierce) and 15-20 ng protein/well were used in ELISAs. All ELISAs were quantified using a microplate reader (FLUOstar Omega). Total (K1503PD-1) and active (K1503OD-1) glucagon-like peptide-1 (GLP-1) in serum was measured with V-PLEX Kits by Meso Scale Diagnostics, and plates were read using the MESO QuickPlex SQ 120 (MSD). All protein concentrations were analyzed as recommended by the manufacturer.

### Statistical analysis

Statistical analyses were performed in GraphPad Prism 9 or JMP (SAS, Cary, NC). Outliers were tested with the Robust Regression and Outlier Removal (ROUT) method (36) and were removed where appropriate. We compared the mouse allergen challenge and surgery groups using parametric or non-parametric tests accordingly (one or two-way ANOVA, Kruskal-Wallis, or t-test), with appropriate post-test, to evaluate significance. PC100 and Reactivity (K) values were determined using non-linear regression with exponential growth curve fit and analyzed with one-tailed t-test with Welch’s correction factor when variances were significantly different as described previously (34).

## Results

### Diet and glucose tolerance

**Figure 2:**
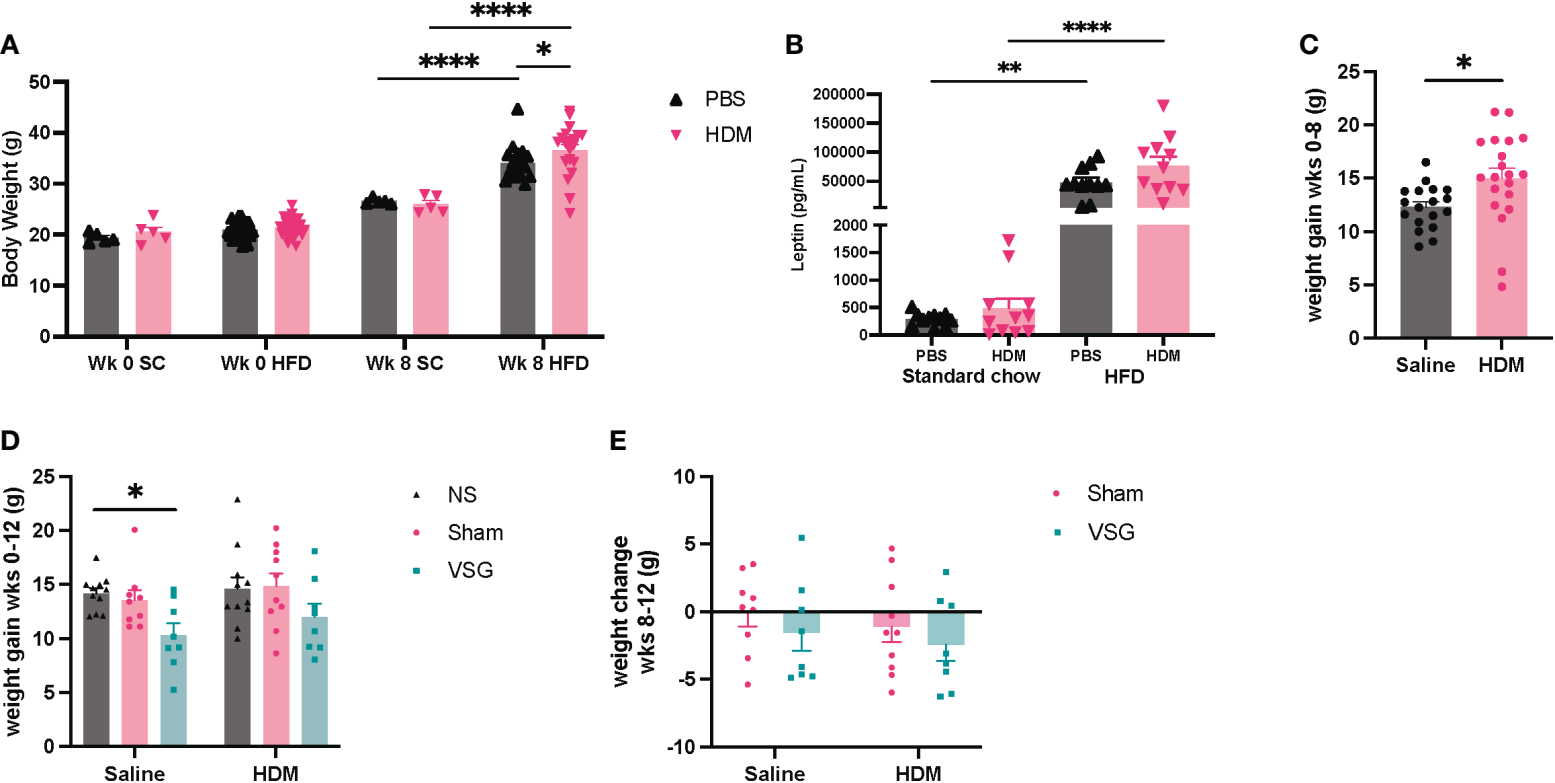
Mouse body weight change. **(A)** Body weight after 8 weeks of HFD or standard chow (SC) diet feeding. **(B)** Serum leptin levels after 8 weeks of HFD or standard chow diet feeding. **(C)** Change in mouse body weight from week (wk) 0 to week 8, n=18-20 mice per group. **(D)** Body weight change in mice from wk 0 to wk 12, n=8-11 mice per group. **(E)** Body weight change from pre-surgery (wk 10) to post-surgery (wk 12), n=8-10 mice per group. **(A, B** were analyzed using a Two-way ANOVA with a Tukey and Sidak *post-hoc* test. **(C)** was analyzed using a non-parametric t-test. **(D, E)** were analyzed using a Two-way ANOVA with a Tukey and Sidak *post-hoc* test. *p<0.05, **p<0.01, ****p<0.0001.

**Figure 3:**
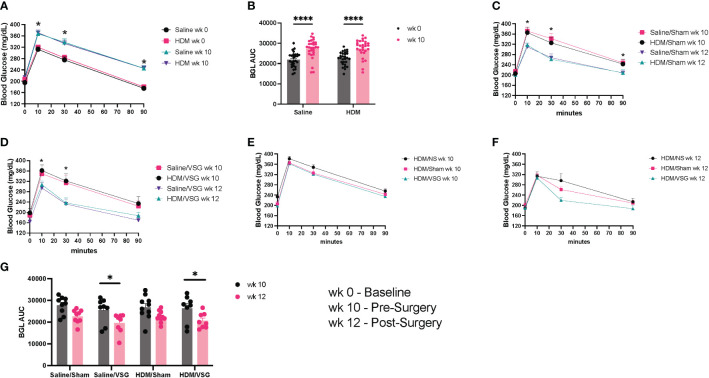
Glucose tolerance. **(A)** Glucose tolerance curves at week (wk) 0 and wk 10, n= 28-29 mice per group. **(B)** Blood glucose area under the curve (AUC) at wk 0 and wk 10. **(C, D)** Glucose tolerance curves at wk 10 and wk 12 for **(C)** Sham (*p<0.05 for HDM/Sham wk 10 vs wk 12 and PBS/Sham wk 10 vs wk 12) and **(D)** VSG mice (*p<0.05 for HDM/VSG wk 10 vs wk 12), n=8-10 mice per group. **(E)** Glucose tolerance curves at wk 10 for HDM-challenged mice. **(F)** Glucose tolerance curves at wk 12 for HDM-challenged mice. **(G)** Blood glucose AUC at wk 10 and wk 12 for Sham and VSG mice. **(A, C–F)** were analyzed using paired t-test and One-way ANOVA and **(B, G)** were analyzed using a Two-way ANOVA with a Tukey and Sidak *post-hoc* test. *p<0.05, ****p<0.0001.

To assess the effects of HFD feeding on body weight and glucose tolerance, mice were weighed and administered an oral glucose tolerance test at the beginning of the protocol, 3 days prior to surgery and again 1-1.5 weeks after surgery. Mice gained weight after 8 weeks of HFD feeding, with increased body weight observed in mice fed the HFD compared to a standard diet for the same time frame (**Figure 2A**). In addition, serum leptin levels were markedly elevated in HFD-fed mice compared to standard chow fed mice after 8 weeks (**Figure 2B**). Greater weight gain was observed in HDM-challenged mice when compared to saline-challenged control mice (**Figure 2C**). Saline/VSG mice had less weight gain over the course of the study when compared to saline/NS mice (**Figure 2D**), with a similar trend seen in the HDM mice. Sham mice did not experience notable weight change when compared to NS mice, and no differences in post-surgical weight change were observed between Sham and VSG mice regardless of challenge group (**Figure 2E**). HFD contributed to glucose intolerance at week 10 for both HDM and saline treated mice (**Figures 3A, B**). Glucose tolerance improved across both procedural groups at week 12 when compared to week 10 measures (**Figures 3C–F**, **Supplementary Figures 1A, B**), with greatest improvement in the VSG mice (**Figures 3F, G**). The mortality rate for Sham and VSG procedures mice was 13.6% and 19.0%, respectively. Taken together, these data show that our model of diet-induced obesity effectively induced weight gain, increased circulating leptin and glucose intolerance in mice, and that VSG improved glucose tolerance in both HDM- and saline-challenged obese mice.

### Lung histology

**Figure 4:**
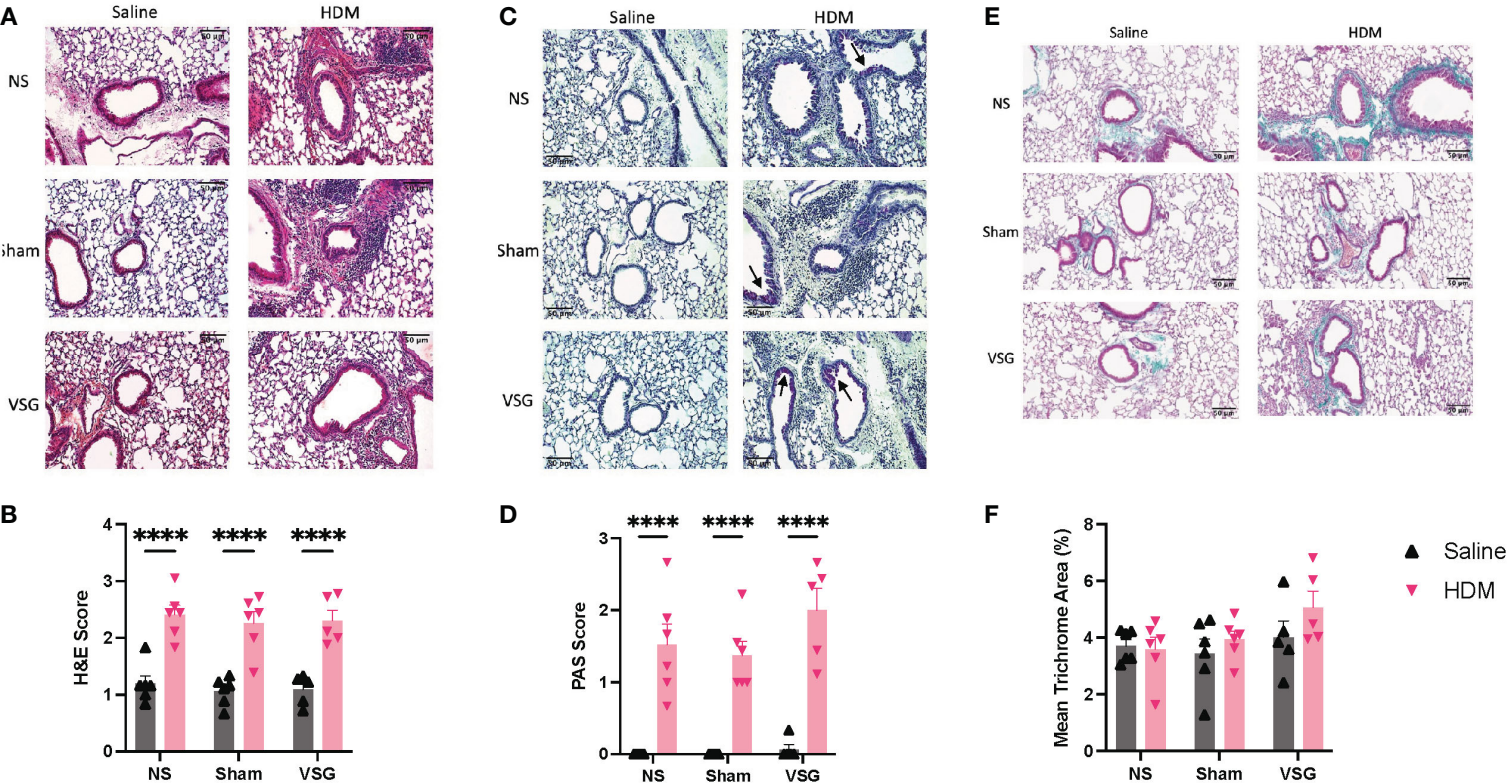
Peribronchial inflammation, mucus and fibrosis. **(A)** Representative images of H&E-stained mouse lung sections taken at x20 magnification, scale bar = 50μm. **(B)** Mean peribronchial inflammation score of H&E-stained lung sections, n=5-6 mice per group, 9-10 airways per mouse. **(C)** Representative images of PAS-stained mouse lung sections taken at x20 magnification, scale bar = 50μm. Arrows indicated positively stained cells in airway epithelium. **(D)** Mean airway mucus score of PAS-stained lung sections, n=5-6 mice per group, 9-10 airways per mouse. **(E)** Representative images of Masson’s trichrome-stained mouse lung sections taken at x20 magnification, scale bar = 50μm. **(F)** Mean percentage peribronchial Masson’s trichrome staining, n=5-6 mice per group, 9-10 airways per mouse. Grey bars and ▲ = saline-challenged mice; pink bars and ▼ = HDM-challenged mice. **(B, D, F)** were analyzed using a Two-way ANOVA with a Tukey and Sidak *post-hoc* test. ****p<0.0001.

Histological assessments of peribronchial inflammation, mucus production and fibrosis as well as airspace inflammatory cell counts were determined at the end of the protocol. H&E- and PAS-stains of mouse lung tissue demonstrated elevated peribronchial inflammatory cell infiltrates and airway epithelium mucus production, respectively, in HDM-challenged mice when compared to saline-challenged control mice (**Figures 4A–D**), consistent with models of allergic airway disease. No difference was observed between surgery groups in either H&E- or PAS-stained airways (**Figures 4A–D**). In HDM-challenged mice, peribronchial collagen deposition had a non-significant increase following VSG (p=0.06) compared to NS mice (**Figures 4E, F**); however, no difference is observed between HDM- or PBS-challenged mice.

**Figure 5:**
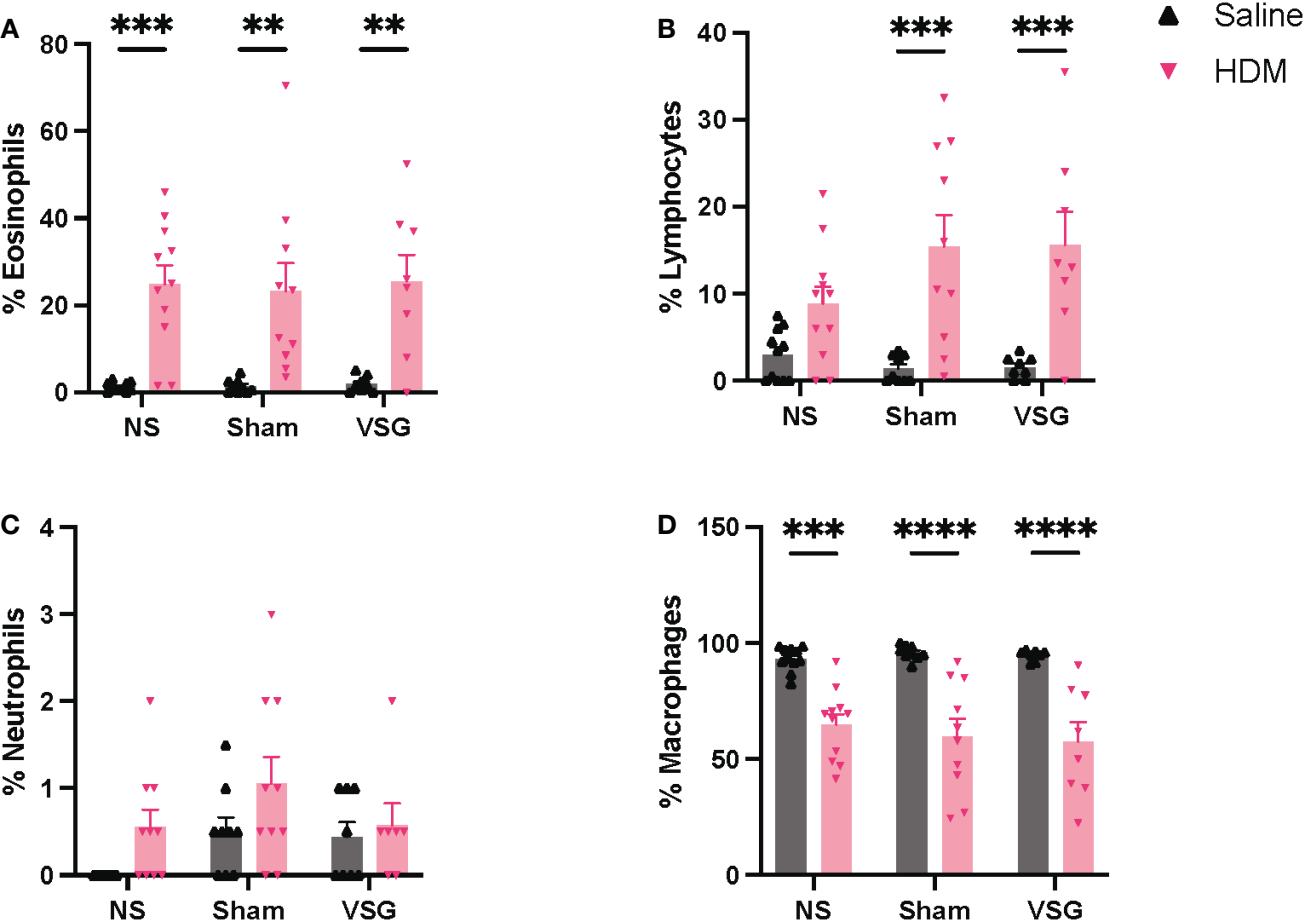
BAL fluid cellularity. Relative percentages of **(A)** eosinophils, **(B)** lymphocytes, **(C)** neutrophils, and **(D)** macrophages in BAL fluid, n=7-11 mice per group. Grey bars and ▲ = saline-challenged mice; pink bars and ▼ = HDM-challenged mice. **(A–D)** were analyzed using a Two-way ANOVA with a Tukey and Sidak *post-hoc* test. **p<0.01, ***p<0.001, ****p<0.0001.

Differential cell counts in BAL fluid are consistent for allergic airway disease, with HDM-challenged mice displaying increased eosinophilia (**Figure 5A**) and reduced macrophages (**Figure 5D**), when compared to saline-challenged mice. HDM-challenged surgery mice also exhibited increased lymphocytes compared to saline-challenged surgery mice, which was not apparent in NS mice (**Figure 5B**). No differences in neutrophil counts were observed in HDM-challenged mice compared to saline-challenged mice (**Figure 5C**). Additionally, no differences were observed for any cell type as a result of Sham or VSG when compared to NS mice in either the saline or HDM challenged groups (**Figures 5A–D**). These histological data demonstrate that our model of chronic allergic airways disease resulted in marked airway eosinophilic inflammation and mucus production, but that no differences were observed between surgical groups, either at baseline or following HDM exposure.

### Lung mechanics

**Figure 6:**
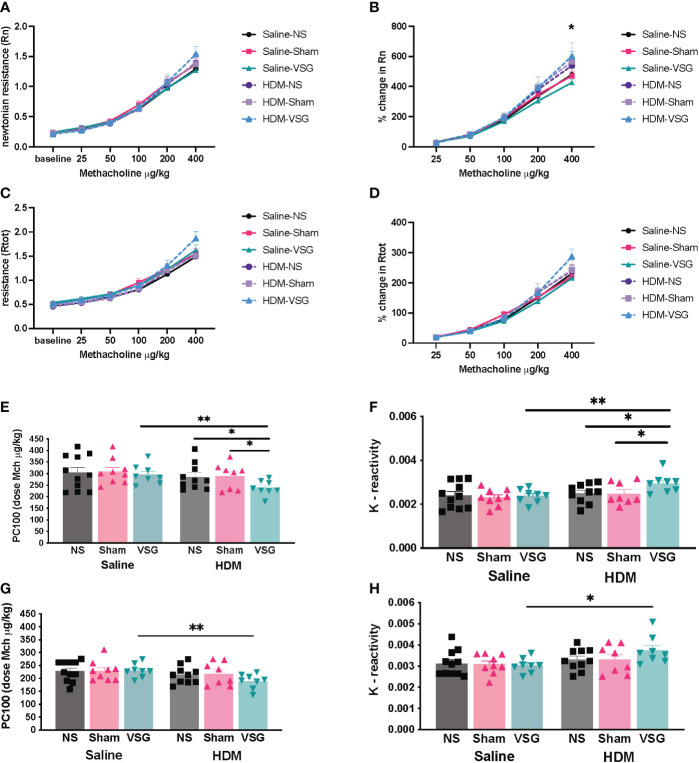
Effects of HDM challenge and VSG on lung mechanics. **(A)** Newtonian resistance [Rn], **(B)** percent change in Newtonian resistance (p<0.05 for HDM/VSG vs Saline/VSG), **(C)** total respiratory resistance [Rtot], and **(D)** percent change in total respiratory resistance with intravenous methacholine challenge, n=8-11 mice per group. **(E)** PC100 for Rtot, n=8-10 mice per group. **(F)** Reactivity [K] for Rtot, n=8-10 mice per group. **(G)** PC100 for Rn, n=8-10 mice per group. **(H)** Reactivity [K] for Rn, n=7-10 mice per group. **(A–D)** were analyzed using repeated measures ANOVA and **(E–H)** were analyzed using one-tailed t-test with Welch’s correction factor. *p<0.05; **p<0.01.

Airway hyperresponsiveness (AHR) is a key feature of allergic asthma pathobiology (37). Obese mice exhibit inherent AHR to bronchocontrictors (38–40). In order to determine the effect of VSG on lung mechanics, mice were assessed *via* Flexivent at 1-1.5 weeks following surgery. We observed that airway responsiveness across all groups of HFD-fed mice increased with increasing doses of methacholine (**Figures 6A, C**). Although HDM challenge did not further increase airway resistance in obese NS mice compared to saline control, the percent change from baseline in Rn was higher in HDM/VSG mice when compared to saline/VSG mice at 400 μg/kg of methacholine (**Figures 6B, D**). In mice challenged with HDM, we observed reduced Rtot sensitivity (PC100) and increased Rtot reactivity (K) with VSG (**Figures 6E, F**), compared to Sham and NS groups, again indicating that VSG enhances airway resistance. Central airway resistance was also augmented in HDM-challenged VSG mice compared to saline-challenged VSG mice, as demonstrated by reduced Rn sensitivity and elevated Rn reactivity (**Figures 6F, G**). No differences were observed between groups with regards to elastance (E), tissue damping (G), tissue elastance (H) (**Supplementary Figure 2**). Taken together, our data provide evidence that at early time points following surgery, VSG stimulates increased total respiratory and central airways resistance in allergic mice.

### Lung tissue mRNA expression

**Figure 7:**
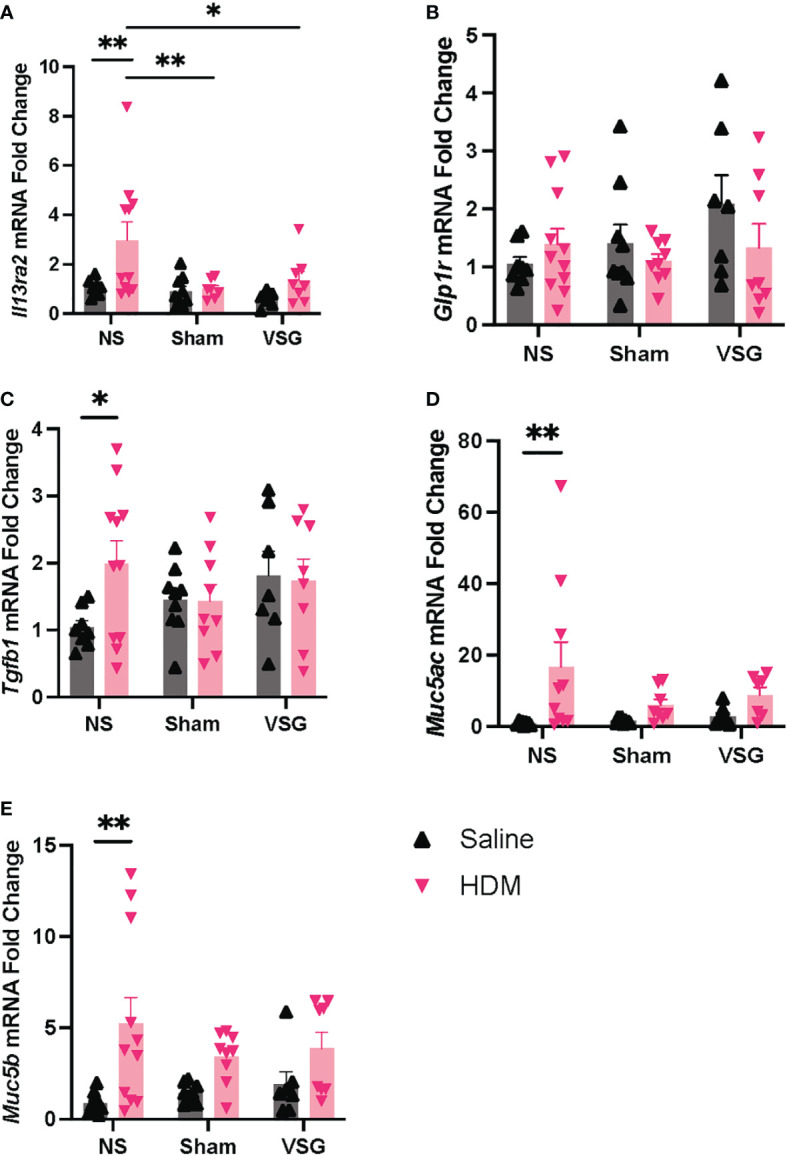
mRNA expression in lung tissue. Lung mRNA expression as measured by quantitative RT-PCR of **(A)** *Il13ra2*, **(B)** *Glp1r*, **(C)** *Tgfb1*, **(D)** *Muc5ac*, **(E)** *Muc5b*, n=6-11 mice per group. Grey bars and ▲ = saline-challenged mice; pink bars and ▼ = HDM-challenged mice. **(A–E)** were analyzed using a Two-way ANOVA with a Tukey and Sidak *post-hoc* test. *p<0.05, **p<0.01.

Quantitative RT-PCR was used to determine mRNA expression changes for genes involved in mediating allergen-induced airway inflammatory and fibrotic responses in lungs of mice in each treatment and surgery group. NS mice challenged with HDM exhibited expected increased expression of *Muc5ac, Muc5b, Tgfb1*, and *Il13ra2* in lung tissue compared to NS saline-treated mice (**Figures 7A, C–E**). Interestingly, both Sham and VSG HDM-challenged mice display decreased lung expression of *Il13ra2* when compared to HDM-challenged NS mice (**Figure 7A**). When compared to NS in saline control mice, VSG exhibited a non-significant increase in expression of lung *Glp1r* (p=0.07) while expression of lung *Glp1r* in HDM-challenged mice is consistent across all surgery groups (**Figure 7B**). Lung mRNA expression of *Il4ra, Il13ra1, Col1a1, Col1a2*, and *Eln* was not different between groups (**Supplementary Figure 3**). Collectively, these data show that interleukin-13 signaling regulation and airway mucin expression may be disrupted in allergic mice with surgery.

### Circulating and pulmonary markers

**Figure 8:**
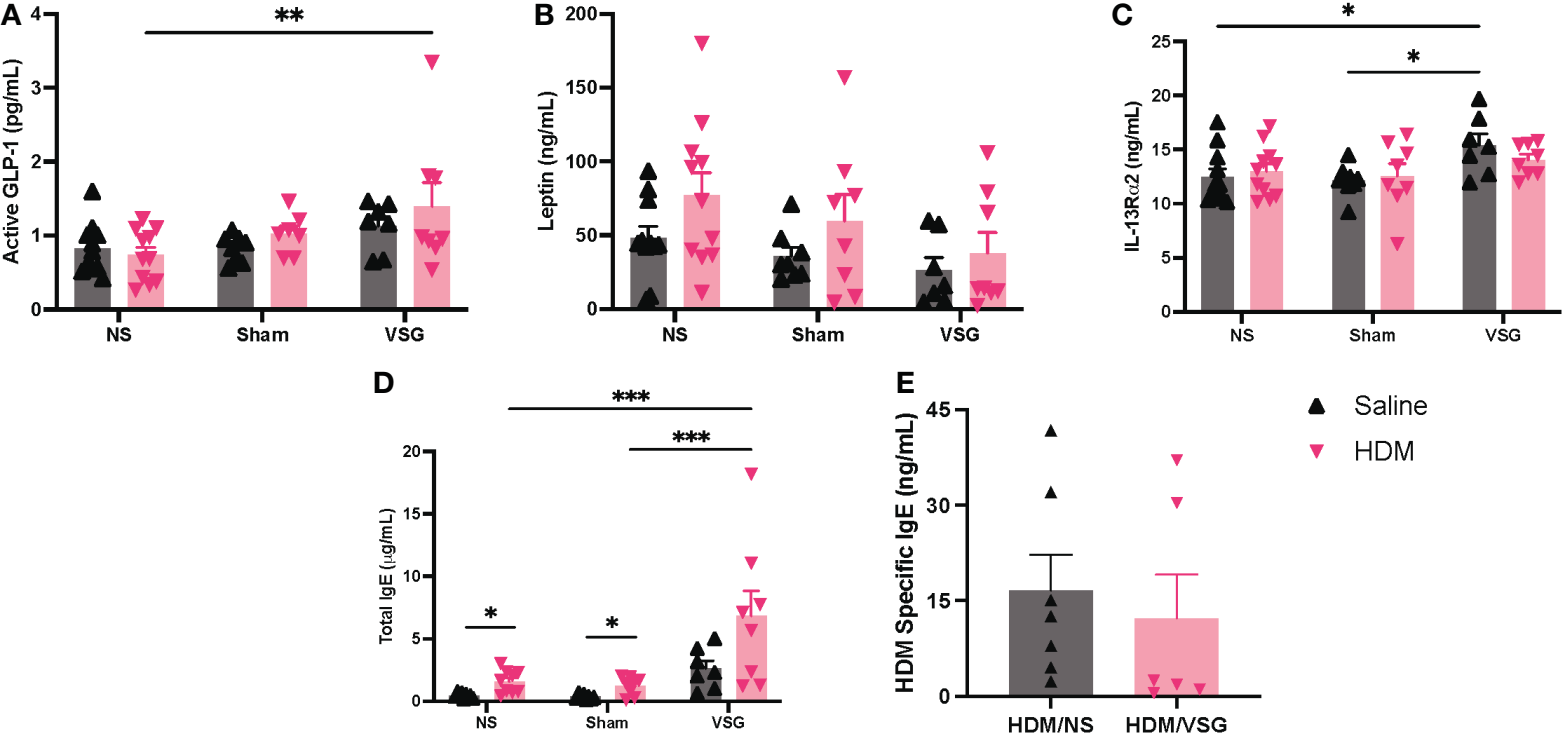
Measurement of cytokines and total and HDM-specific IgE in mouse serum. **(A–E)** Serum concentrations of **(A)** Active GLP-1, **(B)** Leptin, **(C)** IL-13Rα2, **(D)** Total IgE, and **(E)** HDM Specific IgE, n=6-11 mice per group. Grey bars and ▲ = saline-challenged mice; pink bars and ▼ = HDM-challenged mice. **(A–D)** were analyzed using a Two-way ANOVA with a Tukey and Sidak *post-hoc* test. **(E)** were analyzed using a non-parametric t-test *p<0.05, **p<0.01, ***p<0.001.

As allergen and high fat diet feeding as well as VSG can alter circulating and tissue-specific levels of metabolic factors and immune signaling molecules (26, 31), we sought to measure these factors in serum, BAL fluid or lung tissue of mice in our model. Active GLP-1 levels were elevated in serum of HDM-challenged mice that underwent VSG, when compared to the HDM/NS group (**Figure 8A**). Serum leptin levels appeared to be elevated in HDM-challenged mice when compared to saline control across all surgery groups although this effect did not reach significance (**Figure 8B**). However, VSG appeared to reduce serum leptin levels in HDM-challenged mice relative to Sham and NS mice, with a non-significant reduction in VSG mice challenged with HDM compared to HDM/NS mice (p=0.07). In saline treated mice, serum IL-13Rα2 levels were elevated in the VSG group when compared to the NS and Sham groups (**Figure 8C**), while HDM-challenged mice displayed similar IL-13Rα2 concentrations across all surgery groups. As expected, total IgE levels were elevated in HDM-challenged mice relative to saline control mice, characteristic of allergic airway disease (**Figure 8D**). Total IgE was elevated in both groups of VSG mice relative to Sham and NS mice and was further increased in HDM/VSG mice when compared to saline/VSG mice. HDM-specific IgE levels were similar in HDM/VSG mice compared to HDM/NS mice (**Figure 8E**), and undetectable in all groups of saline-challenged mice (data not shown). GLP-1 and IL-13Rα2 protein production was undetectable in all mouse BAL fluid samples (data not shown). Pulmonary levels of total or active TGF-β1, as measured in BAL fluid or lung tissue, were unremarkable between the saline and HDM-challenged mice, irrespective of surgery groups (**Supplementary Figure 4**). Additionally, IL-5, IL-13 and IL-13Rα2 levels, measured in lung tissue, were consistent across all surgical and intranasal treatment groups (**Supplementary Figure 4**). Thus, measurement of serum and pulmonary biomarkers show that allergic responses were induced in all three surgical groups, but that VSG may be involved in modulation of IL-13Rα2 production.

## Discussion

In this study, we investigated the impact of VSG on airway and metabolic physiology, inflammation and fibrosis in a murine model of allergic asthma following chronic HDM challenge. To our knowledge, this study is the first to use a combined chronic allergic airway disease model with obesity and metabolic surgery in experimental mice to assess respiratory disease parameters. Previous studies have investigated the association of bariatric surgery in human patients with allergic asthma (41, 42), or in models of inherent AHR in obese mice, but to date, no studies have been performed in mouse models of chronic allergic airway disease, which allow for analyses of metabolic and inflammatory mechanisms associated with allergen-driven airway pathology. Furthermore, prior VSG studies in mice and rats have shown the influence of this surgery on metabolic physiology, adipose tissue inflammation, gut hormone and adipokine regulation, but these studies did not report features of lung physiology (43–46). Our work provides a foundation for the biological impact of VSG on lung function and airway and systemic inflammatory biomarkers in obese allergic mice.

Obesity was effectively induced in mice after 8 weeks of HFD feeding as demonstrated by elevated body weight and serum leptin levels in HFD-fed mice compared to mice on a normal chow diet for the same time frame, and these observations were valid for both HDM-exposed mice and those administered saline. Additionally, we observed increased body weight gain in HDM-challenged C57BL/6 male mice compared to saline challenged mice after 8 weeks of HFD feeding. Liang et al. made a similar observation in experiments involving chronic administration of ovalbumin to female C57BL/6 mice fed a 60% kcal fat diet for 12 weeks in which the authors reported increased body weight in HFD-fed mice administered ovalbumin compared to mice on HFD alone. Both studies commenced the HFD feeding in 4-5-week-old mice. This intriguing finding that suggests an interaction between allergic exposures and weight gain that may be attributable to alterations of gut microbiota or immune signaling in mice with combined obesity and allergic exposures (47, 48). Future investigations will explore possible mechanisms of increased house dust mite-induced weight gain in obese mice.

The design of our model was intended to reflect acute airway sensitization with a clinically relevant aeroallergen (HDM), followed by chronic (4 week) exposure to the same allergen in the context of obesity. We show that our model of chronic respiratory HDM challenge resulted in the expected robust induction of murine peribronchial inflammation, airway mucus production and eosinophilia, increased serum IgE and lung expression of mucins, *Tgfb1*, and *Il13ra2*, regardless of surgery status. Furthermore, We expected that at the end of this 10-week protocol, these obese mice would exhibit allergen-induced airway inflammation, and indeed our model was effective in eliciting these responses.

The addition of VSG or Sham surgery to the model allowed us to test the hypothesis that VSG would improve features of allergic airway disease in obese mice. At baseline, we observed that VSG mice had increased circulating levels of IL-13Rα2, a negative regulator of type 2 cytokine signaling and allergic asthma responses (49, 50). However, contrary to our hypothesis, we found that AHR to methacholine increased in these mice in the setting of reduced lung tissue expression of *Il13ra2*. A summary of our findings is shown in **Table 1**.

**Table 1:** Summary of Findings.

Measurement	Tissue	VSG vs NS	VSG vs Sham
IL-13Rα2	Serum	↑	↑
*Il13ra2* mRNA	Lung tissue	↓	No change
Total IgE	Serum	↑	↑
Total respiratory resistance (Rtot)	Lung mechanics	↑	↑
Central airway resistance (Rn)	Lung mechanics	↑	No change

Ather et al. showed that sleeve gastrectomy in HFD-fed mice significantly reduced airway responsiveness to methacholine compared to the inherent AHR exhibited by non-surgery HFD-fed mice, driven primarily by changes in distal airway responses (51). Unlike our study, mice were not challenged with allergen. On the other hand, similar to our study, they also found that surgery led to increased airway and systemic biomarkers of inflammation but reduced levels of leptin compared to HFD-fed non-surgery mice. Serum levels of the gut hormone, peptide YY (PYY), were also elevated after the sleeve gastrectomy mice; however, the authors were unable to detect GLP-1 in either BAL fluid or serum. Sham surgery mice were not included in their study. In keeping with our study, their results highlight the impact obesity on airway inflammation and hyperresponsiveness. However, in the present study, we demonstrate that in the presence of hypersensitivity and exposure to inhaled allergens, metabolic surgery may not reduce airway inflammation and AHR in individuals with obesity.

In a study conducted by Dixon et al., asthma patients with obesity who underwent bariatric surgery experienced improved AHR at 12 months post-surgery (41). This improvement was only evident in patients with normal baseline serum IgE levels, and not in patients with atopic asthma and elevated baseline IgE levels, suggesting that the interaction of atopy and obesity impacts the response to surgery in patients with asthma. Our mouse model attempts to recapitulate a phenotype of chronic allergic asthma specific in patients with comorbid obesity; thus, our findings that VSG fails to improve AHR in this model is consistent with the reports that bariatric surgery has differing effects in patients with asthma and comorbid obesity, depending on the underlying asthma phenotype. Indeed, similar to the findings reported by Dixon et al., we observed no improvement in airway resistance following VSG in mice with elevated IgE. An important post-operative complication of vertical sleeve gastrectomy in humans is development of *de novo* gastroesophageal reflux (52), which is a known risk factor for exacerbations associated with airways hyperresponsiveness in asthma (53). It is possible that in our model, VSG contributed to increased gastroesophageal reflux, leading to the observed increased in airways resistance in the mice that underwent VSG. Allen et al. demonstrated that acid aspiration in mice triggered acute AHR, possibly related to increased airway epithelium permeability (54). Although we did not specifically investigate gastroesophageal reflux or airway epithelium permeability in our model, it is plausible that in our model, VSG stimulated acute post-operative acid aspiration that led to increased airway resistance to methacholine.

VSG in rodent models has been used extensively by researchers to study the effects of the procedure on metabolic physiology, including body weight (55), insulin resistance (56), glucose tolerance (57), gastric emptying (46) and central control of satiety (58). VSG also improves hypercapnic ventilatory responses in mice, in a mechanism requiring leptin signaling (59). Our model corroborated that VSG reduced body weight gain and improved glucose tolerance, an effect that was not observed in Sham surgery mice. The lack of specific VSG effects on body weight gain and glucose tolerance may be attributed to the short time of follow-up after surgery at 3 weeks, designed to capture the maximal effects of HDM challenge and better assess the specific effects of VSG while avoiding resolution of airway inflammation that may occur over longer times of follow-up after surgery. Other rodent models (31, 44) that have employed VSG/Sham surgery to investigate glucose tolerance and body weight have reported these measurements at 5-12 weeks after surgery and resumption of HFD feeding, allowing time for the HFD to stimulate weight gain and impaired glucose tolerance in Sham surgery mice. Thus, we speculate that in our model, more time on a HFD following surgery would be needed to demonstrate significant effects of VSG on these features of metabolic disease.

Unique to our study is the investigation of *Il13ra2* expression and production. IL-13Rα2 was originally believed to be a non-signaling decoy receptor which bound IL-13 with high affinity, competing with the IL-4Rα and IL-13Rα1 subunits for IL-13 binding and negatively regulating IL-13 signaling (50). Recent studies suggest its role in development of inflammatory and fibrotic features of disease which may include a capacity to mediate IL-13 signaling (60, 61). Consistent with findings by Piyadasa et al. (62), our study demonstrated higher *Il13ra2* expression in lung tissue in the HDM/NS group when compared to PBS/NS mice. However, expression decreased in HDM-challenged mice that underwent Sham or VSG surgeries, suggesting a surgery effect. On the other hand, baseline levels of circulating soluble IL-13Rα2 were increased in HDM-challenged VSG mice when compared to Sham and NS group, suggesting that VSG elicits changes in *Il13ra2* transcript processing that increase soluble IL-13Rα2 (63) or that changes in *Il13ra2* expression occur in response to the development of airway inflammation. Further studies are warranted to investigate the biological importance of VSG- and allergen-induced changes in IL-13Rα2 expression in obesity.

Our study has several limitations that impact interpretation of the data. As this study aimed to serve as a model of bariatric surgery in human asthma patients, induction of obesity in mice using a HFD was required; thus, no comparisons with lean mice were included in the study. Also, only male mice were used for this study as male C57BL/6J mice are more susceptible to weight gain on a HFD than female mice (29). Sex differences may be important in this setting as asthma, in general, as well as obesity-associated asthma is more prevalent in adult females than males (2, 64). More studies using female mice in a mixed sex study to compare responses to male mice are needed to define sex differences using our model. In addition, collection of tissues and lung mechanics measurements was done at a short follow-up time after surgery when some metabolic effects were not as yet evident. Future work including both sexes, as well as in other strains of mice (e.g. Balb/c (65)) would help validate our counter-intuitive findings and provide a better approximation of how bariatric surgery impacts features of asthma in patients with obesity. Additionally, conducting the experiment across longer timepoints following surgery, with increased chronic exposure to allergen challenge and HFD, will give a better understanding of specific changes in physiology (e.g. glucose tolerance) and how these impact allergic airway disease.

In conclusion, the current study demonstrates that VSG in a murine model of chronic allergic airway disease increases airway resistance in the short term following surgery and alters IL-13Rα2 expression. This study offers insight as to the mechanisms governing effects on airway pathobiology following bariatric surgery in patients with allergic asthma and comorbid obesity. Further studies of allergic airway disease in experimental mice employing VSG that investigate the long-term impacts of surgery in the context of obesity are warranted.

## Data availability statement

The original contributions presented in the study are included in the article/**Supplementary Material**. Further inquiries can be directed to the corresponding author.

## Ethics statement

The animal study was reviewed and approved by Duke University Institutional Animal Care and Use Committee.

## Author contributions

JW, methodology, investigation, validation, formal analysis, and writing - original draft. MI, methodology, formal analysis, investigation, writing- review, and editing. VM and AH, methodology, investigation, writing- review, and editing. MM, RT, and LQ, methodology, writing- review, and editing. SP, writing- review, and editing. JW, methodology, formal analysis, writing- review, and editing. JI, conceptualization, methodology, investigation, resources, writing - review and editing, and funding acquisition. All authors contributed to the article and approved the submitted version.
