# Supplementary material for: Vertical sleeve gastrectomy associates with airway hyperresponsiveness in a murine model of allergic airway disease and obesity
Source: Front Endocrinol (Lausanne). 2023 Feb 28;14:1092277. doi: 10.3389/fendo.2023.1092277 (PMC10011633; doi:10.3389/fendo.2023.1092277)
Supplement: Supplementary file 1 [file DataSheet_1.pdf]

ONLINE DATA SUPPLEMENT

Figure Legends

Supplemental Figure 1. Glucose tolerance. (**A**) Glucose tolerance curves at wk 10 for saline-challenged mice. (**B**) Glucose tolerance curves at wk 12 for saline-challenged mice. **A,B** were analyzed using One-way ANOVA.

Supplemental Figure 2. Lung mechanics. **(A)** elastance [E], **(B)** percent change in elastance, **(C)** tissue damping [G], **(D)** percent change in tissue damping, **(E)** tissue elastance [H], and **(F)** percent change in tissue elastance with intravenous methacholine challenge, n=7-11 mice per group. **A-F** were analyzed using repeated measures ANOVA.

Supplemental Figure 3. mRNA expression in lung tissue. Lung mRNA expression as measured by quantitative RT-PCR of **(A)** *Il4ra*, **(B)** *Il13ra1*, **(C)** *Col1a1*, **(D)** *Col1a2,* and **(E)** *Eln*, n=6-11 mice per group. Grey bars and ▲ = saline-challenged mice; pink bars and ▼ = HDM-challenged mice. **A-E** were analyzed using a Two-way ANOVA with a Tukey and Sidak post-hoc test.

Supplemental Figure 4. Changes in inflammatory biomarkers in BAL fluid or lung tissue. **(A)** Total TGF-β1 in BAL fluid, n=7-11 mice per group. **(B-F)** Concentration of **(B)** Total TGF-β1, **(C)** Active TGF-β1, **(D)** IL-5, **(E)** IL-13, and **(F)** IL-13Rα2 in lung tissue, n=7-11 mice per group. Grey bars and ▲ = saline-challenged mice; pink bars and ▼ = HDM-challenged mice. **A-D** were analyzed using a Two-way ANOVA with a Tukey and Sidak post-hoc test. **E** were analyzed using a non-parametric t-test.
