# Supplementary figures and images for: Vertical sleeve gastrectomy associates with airway hyperresponsiveness in a murine model of allergic airway disease and obesity
Source: Front Endocrinol (Lausanne). 2023 Feb 28;14:1092277. doi: 10.3389/fendo.2023.1092277 (PMC10011633; doi:10.3389/fendo.2023.1092277)

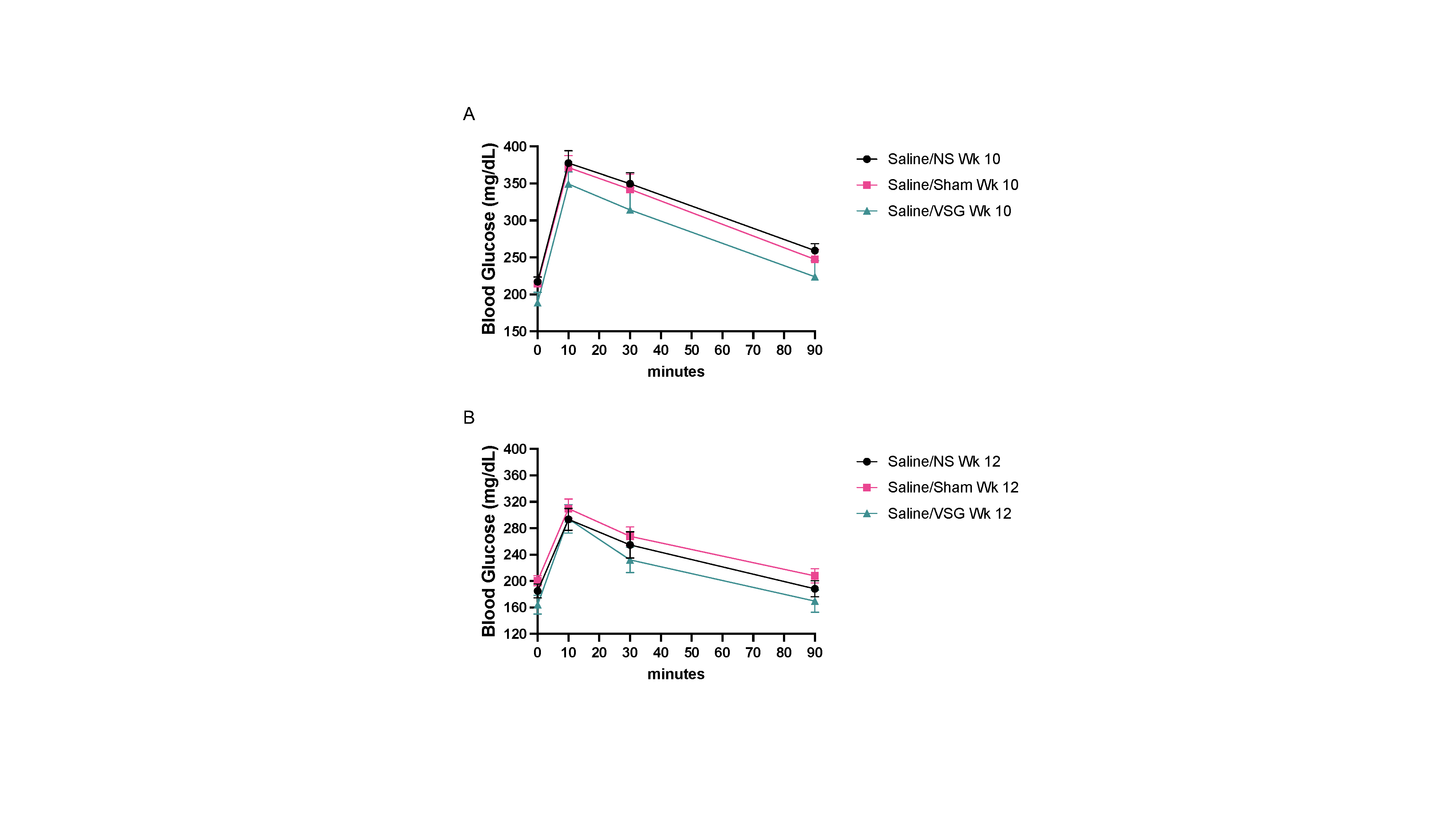

Supplement: Supplementary file 2 [file Image_1.tiff]

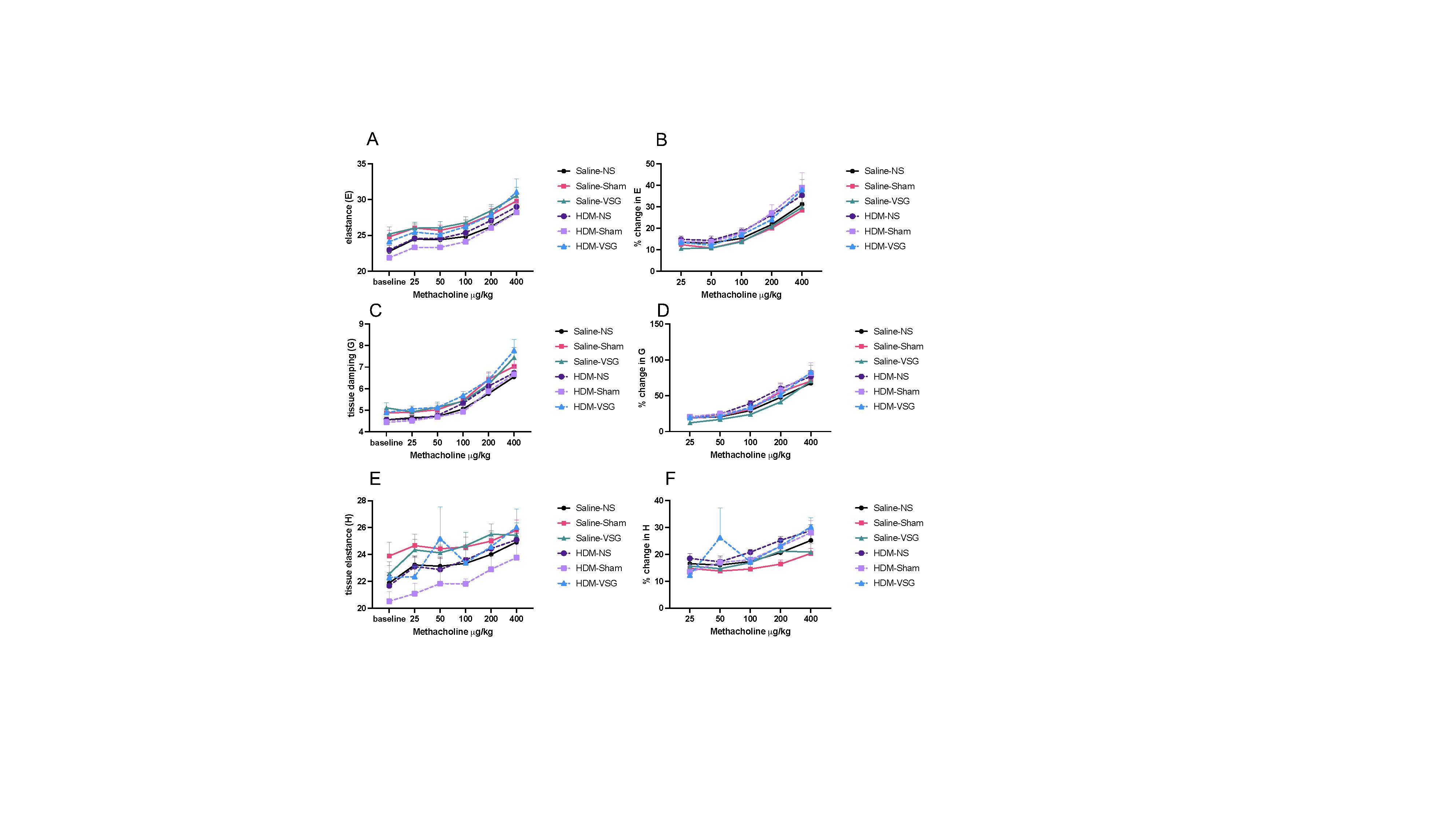

Supplement: Supplementary file 3 [file Image_2.tiff]

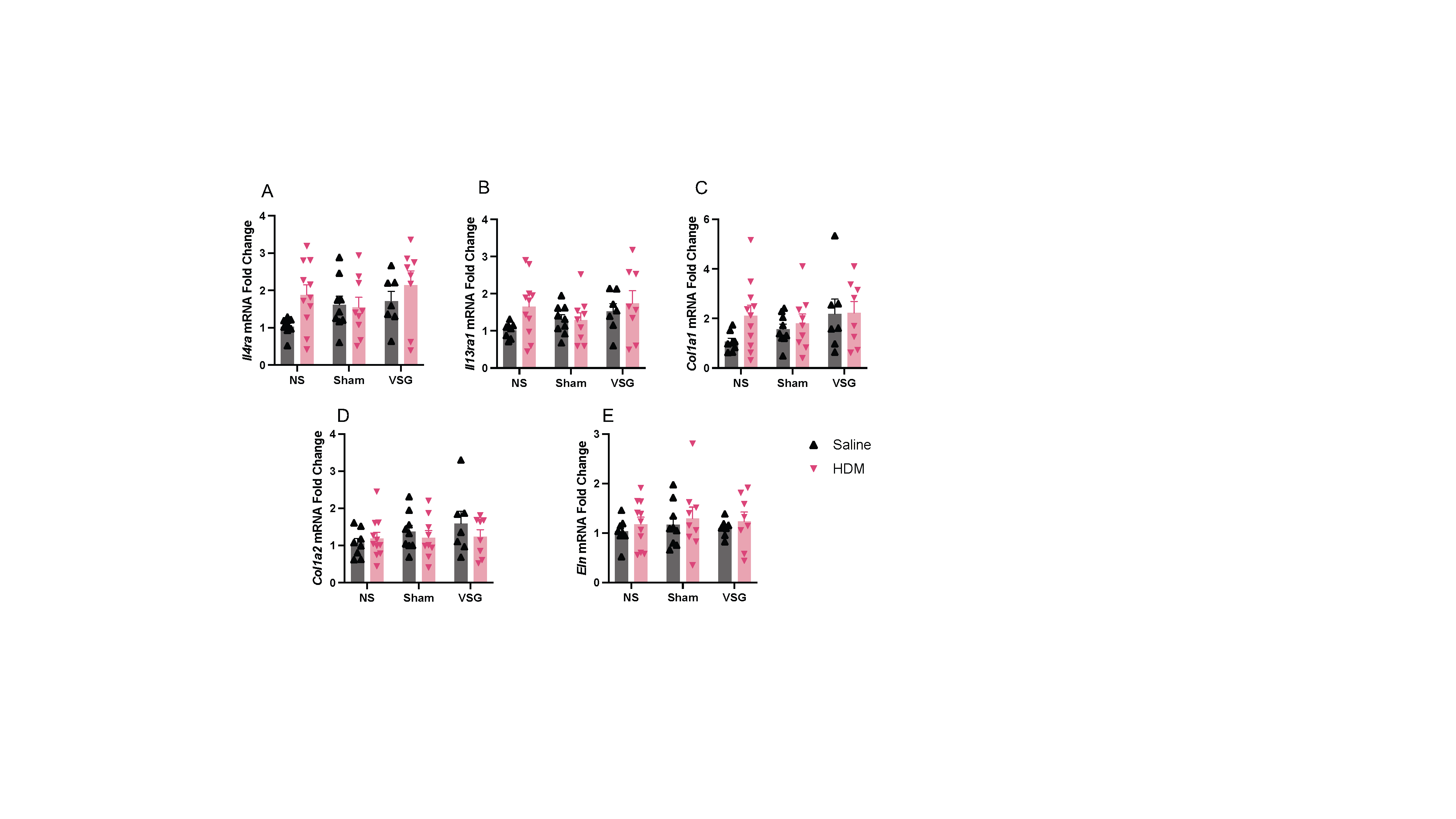

Supplement: Supplementary file 4 [file Image_3.tiff]

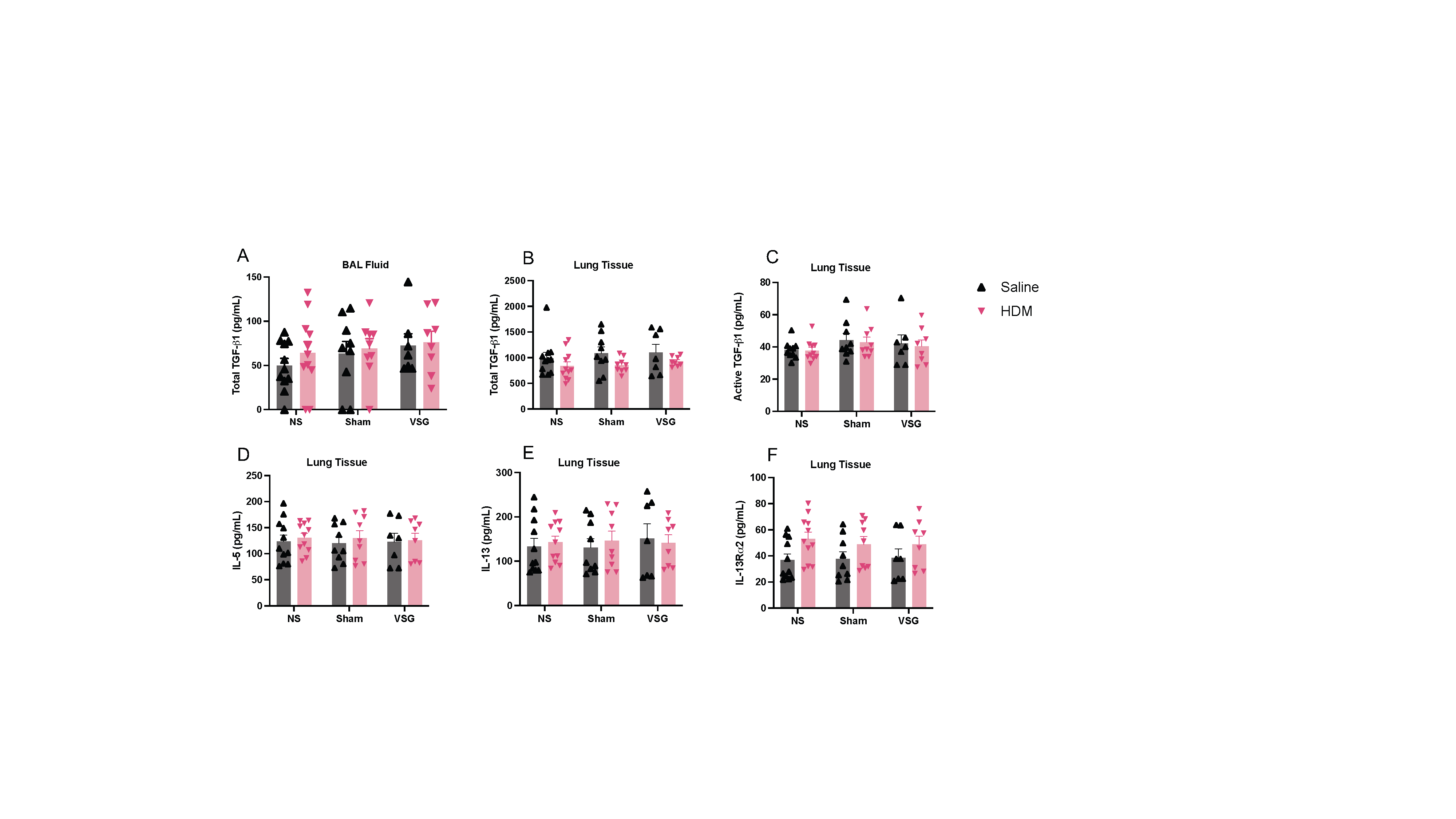

Supplement: Supplementary file 5 [file Image_4.tiff]
